# GILZ as a Regulator of Cell Fate and Inflammation

**DOI:** 10.3390/cells11010122

**Published:** 2021-12-30

**Authors:** Stefano Bruscoli, Carlo Riccardi, Simona Ronchetti

**Affiliations:** Department of Medicine and Surgery, Section of Pharmacology, University of Perugia, 06132 Perugia, Italy; stefano.bruscoli@unipg.it (S.B.); simona.ronchetti@unipg.it (S.R.)

**Keywords:** glucocorticoid, GILZ, inflammation, immune system

## Abstract

One of the human body’s initial responses to stress is the adrenal response, involving the release of mediators that include adrenaline and glucocorticoids (GC). GC are involved in controlling the inflammatory and immune response mechanisms. Of these, the molecular mechanisms that contribute to anti-inflammatory effects warrant more investigation. Previously, we found that GC induced GILZ (glucocorticoid-induced leucine zipper) quickly and widely in thymocytes, T lymphocytes, and other leukocytes. GILZ regulates the activation of cells and is an essential mediator of endogenous GC and the majority of GC anti-inflammatory effects. Further research in this regard could lead to the development of an anti-inflammatory treatment that yields the therapeutic outcomes of GC but without their characteristic adverse effects. Here, we examine the mechanisms of GILZ in the context of GC. Specifically, we review its role in the proliferation and differentiation of cells and in apoptosis. We also examine its involvement in immune cells (macrophages, neutrophils, dendritic cells, T and B lymphocytes), and in non-immune cells, including cancer cells. In conclusion, GILZ is an anti-inflammatory molecule that could mediate the immunomodulatory activities of GC, with less adverse effects, and could be a target molecule for designing new therapies to treat inflammatory diseases.

## 1. Introduction

The stress response is an important complex of mechanisms aimed at regulating numerous body functions essential for survival. When the body perceives stress stimuli, a series of immediate and/or longer-term responses begins. Among the first responses is the adrenal response, involving the production of a series of mediators, including adrenaline and glucocorticoids (GC) released from the adrenal cortex. These are important mediators that can affect the many functions of the organism by acting on practically all tissues. For example, these range from the nervous system to the immune system, from metabolic events and pressure control to the regulation of cell growth and survival, from behavioral aspects to learning and memorization skills. Notably, GC control the mechanisms of the inflammatory and immune responses. While the majority of GC effects on cells and tissues are relatively well known, the molecular and cellular mechanisms, particularly the molecular mechanisms contributing to the anti-inflammatory effects, require further study.

A number of years ago, we began a research approach aimed at discovering transcriptional products consequent to GC interaction with glucocorticoid receptors (GR). In the present study, we identified transcripts that included many different components of the proliferation, cell death, immune, and inflammatory systems. The experimental approach was conducted by treating thymocytes or a T cell line (3DO) in vitro. We found that some transcripts were differentially expressed in dexamethasone (DEX)-treated cells. Among the many molecules, we focused on two: GILZ (glucocorticoid-induced leucine zipper), in treated thymocytes, and GITR (glucocorticoid-induced TNF receptor superfamily related gene), in the 3DO cell line. Subsequent experiments confirmed that GC treatment induced GILZ rapidly and widely in many cell types in vivo, including thymocytes, T lymphocytes, and macrophages, but not GITR [1]. GITR is not up-regulated in normal cells in vivo, although it is normally expressed in many cells and tissues, including, for example, lymphocytes, keratinocytes and neurons [2,3] and plays a role in infections and the inflammatory/immune processes.

It was clear from the outset that, as indicated in the first published paper on GILZ [4,5], GILZ regulates cell activation, including that of T cells; therefore, it is an endogenous regulator of the inflammatory/immune processes. Moreover, other than being an important mediator of endogenous GC, GILZ mediates most of the anti-inflammatory effects of exogenous GC, which could give rise to new anti-inflammatory treatments that mimic the curative effects of GC while not inducing typical GC adverse effects. These detrimental effects limit any chronic GC therapy, as first shown in different inflammatory colitis models [6].

## 2. GILZ in Controlling Cell Growth: Effect on Apoptosis, Cell Proliferation and Differentiation

Since its discovery, it was clear that GILZ can control cell survival and in particular can counter T cell death as a consequence of T cell activation [4]. In this respect, GILZ can inhibit cell activation and the consequent cell death, thereby favoring the survival of antigen (T cell receptor, TCR)-stimulated lymphocytes, which then become anergic, inhibiting the inflammatory/immune response. Like GC, GILZ counters TCR-mediated activation-induced apoptosis but inhibits the development of an immune response. Shared by GILZ and GC, this effect correlates with the inhibition of interleukin (IL)-2 production and the consequent upregulation of Fas and FasL in T cells.

Notably, GC counter T cell death consequent to cell activation, but also function as apoptosis inducers, which is appropriate for a stress-induced response aimed at sparing cells that can be saved while favoring the elimination of heavily damaged cells. Similarly, GILZ induces apoptosis in non-activated thymocytes, and like GC, activates caspases important in cell death induction [7,8,9,10]. 

GC also exert an anti-proliferative effect that can be considered part of the anti-inflammatory and immunosuppressive functions. Similar to GC, GILZ also exerts an anti-proliferative effect and inhibits T cell proliferation consequent to TCR stimulation, counteracting the clonal expansion typical of the T cell immune response important for amplifying antigen-specific T cell clones and memory T cell generation [11,12].

GILZ protein contains in its central part a canonical leucine zipper domain but, differently from most leucine zipper proteins, GILZ lacks a basic domain necessary to bind DNA and there are no canonical DNA-binding or nuclear localization signal domains, thus it probably does not act as a direct transcription factor as the majority of other members of leucine zipper family. Its highly conserved leucine zipper domain is necessary not only to form homodimers but also to heterodimerize with other proteins of the same family. Thus far, GILZ has been mostly localized in the cytoplasm, and most of the GILZ immunosuppressive and anti-inflammatory effects are associated with inhibition of signal transduction pathways localized in the cytoplasm, such as the mitogen-activated protein kinase (MAPK) inhibition capability, common to GILZ and GC. Notably, the anti-proliferative effect and apoptosis induction of GC are significantly weakened in the absence of GILZ, indicating that GILZ mediates the GC growth inhibitory effects.

At the embrional level, GC regulate cell differentiation and function as morphogenic agents, particularly for tissues such as the lung and intestine [13]. GILZ also plays a role in cell differentiation in different cells and tissues, including T lymphocytes, myocytes, and spermatocytes, as described in detail below. Notably, GILZ is important for generating T cell subpopulations such as regulatory T cells (Tregs), mediating the GC promoting activity in Treg generation [14]. Moreover, similarly to GC, GILZ stimulates type 2 T helper (Th2) differentiation, and this effect was curative in an inflammatory experimental disease of the lung, the first disease model in which GILZ overexpression has been shown to exert a therapeutic effect [15].

## 3. GILZ Function in Macrophages

A pioneer study on innate cells first described GILZ induction by GC in human monocytes/macrophages [16]. Subsequently, Berrebi and colleagues demonstrated constitutive GILZ expression in macrophages from various human tissues, from the glomeruli in the kidney to the Kupffer cells of the liver. In these macrophages, GC and IL-10 upregulated GILZ, as subsequently demonstrated in other cell types. In Crohn’s disease, GILZ was found only in macrophages from tissue surrounding the granulomas, but not internally, where macrophages were in the active state. The same group observed GILZ expression in macrophages with phagocytic activity in Burkitt lymphoma [17]. The authors observed an interesting correlation with the activation state of macrophages and poor or null GILZ expression, pioneering what other studies confirmed later in neutrophils [18].

GILZ has been identified as a target of the annexin A1 (ANXA1) anti-inflammatory effect. ANXA1 (or lipocortin) is a classical anti-inflammatory factor induced by GC whose regulation is still not completely understood [19,20]. Interestingly, reciprocal regulation of ANXA1 and GILZ has been demonstrated in macrophages and neutrophils. In the former, GC barely induce GILZ in the absence of ANXA1, whereas, in the latter, GC cannot upregulate ANXA1 in the absence of GILZ, as described above. Specifically in macrophages, ANXA1 negatively regulates tumor necrosis factor α (TNF-α) and IL-6 through GILZ, which in turn prevents activation of the extracellular signal-regulated kinase (ERK)-MAPK pathway, rendering macrophages unresponsive to lipopolysaccharide (LPS) stimulation [21]. Indeed, macrophages use LPS tolerance as a mechanism for surviving a second wave of LPS stimulation after an initial inflammatory immune response, especially during the late phase of sepsis [22]. Further, in human alveolar macrophages, GILZ is downregulated by LPS-dependent stimulation of Toll-like receptors (TLRs) via MyD88 (innate immune signal transduction adaptor), which delivers intracellular signals leading to the release of pro-inflammatory factors [23]. GILZ downregulation therefore represents a mechanism by which macrophages can increase phagocytosis and killing ability against infective agents [24]. GILZ involvement in the response to LPS has been assessed by the observation of high GILZ expression levels in macrophages of the SPRET/Ei mouse strain, an inbred LPS-resistant strain [25]. All these examples have presented evidence that GILZ is both a central endogenous protein for controlling excessive inflammatory response and a mediator of GC anti-inflammatory effect in these cells [26]. Interestingly, GILZ can be induced in macrophages by curcumin, a known natural anti-inflammatory compound, by enhancing the stability of its mRNA [26]. Therefore, GILZ strongly contributes to the establishment of a tolerogenic state in macrophages, limiting inflammation and mirroring what has been observed in dendritic cells (DC), as described below.

**Figure 1:**
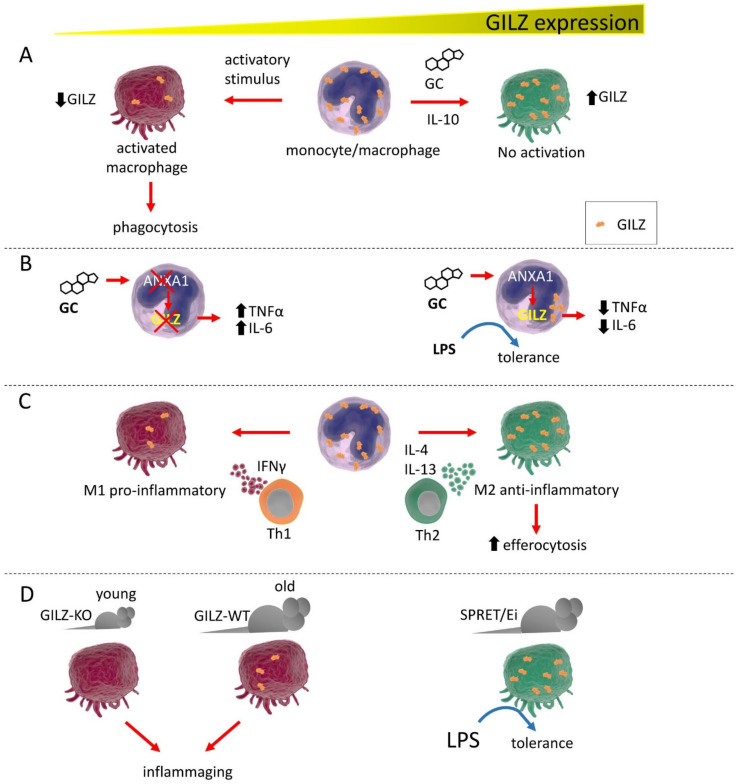
GILZ expression correlates with monocyte/macrophage activation state and functions. (**A**). GC or IL-10 induce high GILZ expression levels, inducing the resting state in these cells. Low GILZ expression allows macrophages to activate and initiate phagocytic activity. (**B**). GILZ is a target of ANXA1 and cannot be fully induced by GC in the absence of ANXA1, with subsequent release of proinflammatory cytokines. Conversely, ANXA1 induction favors GILZ expression, driving macrophages to a state of tolerance, reducing proinflammatory cytokine expression, and inducing unresponsiveness to LPS exposure. (**C**). Depending on the stimulating cytokines released by Th-specific cells, macrophages exist under two distinct phenotypes: the proinflammatory M1 and the anti-inflammatory M2. GILZ expression is low in M1 macrophages and is high in M2 cells, with consequent raised capability for efferocytosis. (**D**). GILZ ablation in GILZ-KO mice confers an activated phenotype to the macrophages of young mice that is very similar to those of old WT mice, which are macrophages prone to causing inflammation, i.e., inflammaging. In the LPS-resistant SPRET/Ei mouse strain, GILZ is expressed at abnormally high levels in macrophages, which show a tolerogenic phenotype.

Although the classical dichotomy model of activated macrophages has been recently replaced by the newly identified states of activation, GILZ has been found to be preferentially expressed in the previously so-called M2 macrophages, cells with an anti-inflammatory role. Conversely, GILZ is poorly expressed or even absent in M1-like cells. In addition, GILZ enhances efferocytosis, the process of engulfing apoptotic cells in an inflammatory context. Therefore, GILZ not only helps reduce inflammation but also contributes to the resolution process of inflammation and, in the future, a correlation of GILZ expression with the diverse states of macrophage activation and their induction by the micro-environmental milieu will be worth deep investigation (Figure 1) [27]. Very recently, GILZ induction in monocytes/macrophages in a mouse model of septic shock was sufficient to hamper the systemic inflammatory response in vivo while preventing bacterial spread, suggesting a protective role for GILZ in maintenance of a reduced general inflammatory state [28]. Whether the sole regulation of GILZ expression in macrophages is sufficient for maintaining a general reduced inflammatory state remains to be investigated, considering that GILZ expression in other immune cells also contributes to counteracting inflammation. Another finding that supports this correlation of low GILZ expression with inflammation is from studies on aged mice, whose macrophages showed the same features as those of young GILZ knock-out (KO) mice, favoring inflammaging, a general low and chronic inflammatory state of the immune system in the elderly [29].

As macrophages are a cell type that basally express GILZ, a pivotal role for GILZ in controlling their activation state has emerged over the years, rendering GILZ a possible target for innate immunity-centered therapies.

## 4. GILZ Function in Neutrophils

Together with macrophages and DC, neutrophils represent the pillar of the innate immune response. Recent and scarce studies have shown a critical function of GILZ in these cells, in the control of both their activation state and survival. Our group demonstrated that GILZ is necessary for preventing excessive neutrophil activation, both in vitro and in vivo, in mouse models of *Candida albicans* infection and acute colitis. Neutrophils from GILZ-KO mice displayed a more activated phenotype, whereas wild-type (WT) cells showed a lower degree of activation because of MAPK pathway inhibition by GILZ [18]. GILZ therefore functions as a brake in these cells, consistent with reports on macrophages. On the other hand, GILZ over-expression in a human cell line that differentiated towards the neutrophil lineage promoted apoptosis, with JNK pathway activation and downregulation of the anti-apoptotic factor myeloid cell leukemia-1 (Mcl-1) [30].

Beginning in the late 1990s, GILZ has been reported to mediate several GC anti-inflammatory and immunosuppressive effects in many cell types. Neutrophils represent the most recent immune cell type in which GC have been demonstrated to exert their functions via GILZ induction. ANXA-1, one of the most efficient anti-inflammatory and pro-resolving proteins induced by GC, was newly transcribed in response to steroid treatment only after GILZ induction. This effect was supported by the observation that GILZ depletion rendered GILZ-KO neutrophils incapable of upregulating ANXA-1, a pivotal protein for GC to prevent neutrophil migration into inflamed tissues. ANXA-1 is induced by GILZ via binding to PU.1, a transcription factor that is a negative regulator of *ANXA-1* gene transcription [31].

In neutrophils infiltrating the alveoli of patients with acute respiratory distress syndrome (ARDS), GILZ was upregulated in about 75% of the corticosteroid-treated patients, in an attempt to counteract excessive inflammation. In addition, the high level of GILZ mRNA expression was correlated to more severe disease. Interestingly, that study determined that GILZ expression was a transient phenomenon, given the transcripts’ half-life around 2 h [32].

**Figure 2:**
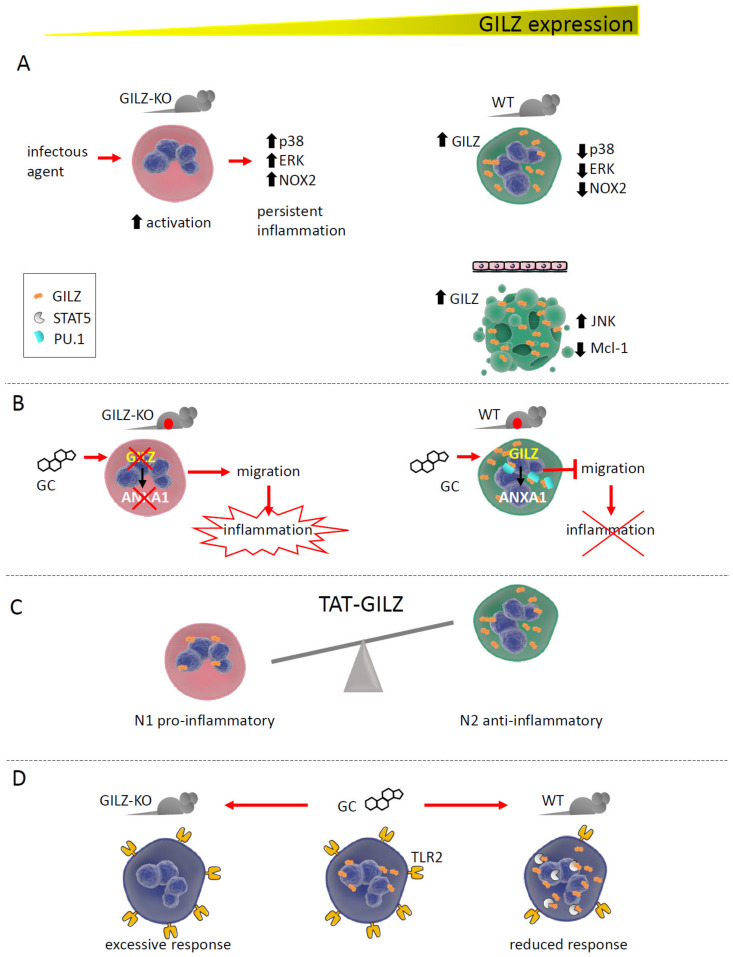
GILZ expression correlates with neutrophil activation state and functions. (**A**). GILZ ablation in GILZ-KO mice causes excessive neutrophil activation in response to inflammatory stimuli. Conversely, in WT mice, GILZ upregulation after activation inhibits the MAPK pathway and NOX2 (NADPH oxidase 2), reducing neutrophil activity. In a human cell line, GILZ upregulation caused apoptosis by upregulating JNK and downregulating Mcl-1. (**B**). The absence of GILZ in GILZ-KO mice with acute peritonitis prevents GC-dependent ANXA1 expression, whereas WT mice can express ANXA1, blocking neutrophil migration into inflamed tissues. By binding PU.1, GILZ promotes *ANXA1* gene transcription. (**C**). GILZ treatment favors the development of N2 anti-inflammatory neutrophils in a model of AKI. (**D**). In vivo treatment with GC upregulates GILZ in circulating neutrophils, favoring its binding with STAT5 and downregulating TLR2 expression. The absence of GILZ maintains TLR2 levels, causing an excessive neutrophil response.

Similarly to macrophages, in which selective GILZ expression is unique to a specific subtype, GILZ in neutrophils differently regulates the N1 (pro-inflammatory) and N2 (anti-inflammatory) cell types. In a model of acute kidney injury (AKI), treatment with the recombinant protein TAT-GILZ promoted the development of N2 neutrophils with the suppressive phenotype, restoring the N1/N2 balance while favoring renoprotection (Figure 2) [33].

A protective role of GILZ in neutrophil function is gradually emerging, which may have implications in neutrophil dysfunction-based diseases [34].

## 5. GILZ Function in Dendritic Cells

DC determine T lymphocyte activation in response to antigen-presenting cell (APC)-dependent antigen presentation. Since the first studies on DC, GILZ has emerged as a protein with an active function in driving DC towards a tolerogenic phenotype. In these cells, GILZ is induced not only by GC, but also by IL-10 and transforming growth factor β (TGFβ), two immunosuppressive cytokines, and by vitamin D3, mitomycin C, rapamycin, and hepatocyte growth factor [35,36]. In addition, tolerogenic DC can themselves release IL-10 and TGFβ, strengthening the suppression loop of immune activation. GILZ seems therefore to be at a crossroads where GC and immunosuppressive cytokines contribute to counteracting inflammation. Mechanistically, GILZ expression in DC avoids antigen presentation to T lymphocytes, thereby preventing their activation [37]. Furthermore, besides inducing the expression of tolerizing molecules such as those described above, GILZ can prevent on DC the expression of CD80 and CD86, T cell-stimulating molecules while simultaneously suppressing pro-inflammatory cytokine production. As a consequence of these immunosuppressive effects, GILZ-expressing DC become immature and tolerogenic, directing T cells towards the regulatory phenotype. This mechanism contributes to the immunosuppressive function of GC treatment in DC and to natural control of inflammation by endogenous GC [38,39]. On the other hand, pharmacological silencing of GILZ with small interfering RNA (siRNA) activates DC by increasing IL-12 secretion and reducing programmed cell death ligand 1 (PD-L1) expression, favoring T cell activation. This approach holds promise for generating functional DC-based cancer vaccines [40].

DC also play important roles also in allergic diseases. A study of respiratory allergic patients found that GILZ mediates GC efficacy, as steroid treatment restored GILZ expression to normal levels in DC isolated from peripheral blood, inducing a tolerant phenotype in DC and activating IL-1-Tregs [41].

GILZ controls another important function of DC, macropinocytosis, one of the mechanisms by which DC acquire antigens. In CD8α+ DC, the subset in which GILZ is mostly expressed, micropinocytosis is very efficiently inhibited by GILZ, whereas GC enhance antigen capture. Reduced micropinocytosis affects the ability of DC to fully activate CD8+ T cells [42].

**Figure 3:**
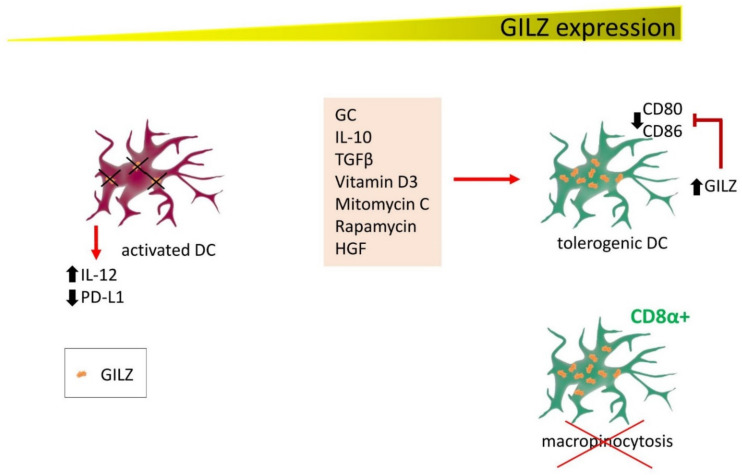
GILZ expression correlates with DC activation state and functions. Several factors induce GILZ expression in DC, contributing to driving DC towards a tolerogenic state. GILZ decreases CD80 and CD86 expression, reducing their capability for activating T cells. Conversely, DC are activated in conditions of low or null GILZ expression (either by experimentally inducing GILZ silencing or in patients with respiratory allergic disease), releasing IL-12 and reducing PD-L1 expression. In addition, CD8α+ cells, the DC phenotype with the highest GILZ levels, lose the ability to exert micropinocytosis.

Overall, these studies identify an important function of GILZ in DC (Figure 3), as the immune response in the entire body depends on how efficient APCs are in presenting the antigen. When this process fails, DC become tolerogenic and no response by T cells can occur, contributing, in the worst case, to a favorable microenvironment for tumor development. In other contexts, the induction of tolerogenic DC is required to dampen inflammation or autoimmune diseases.

## 6. GILZ Function in T Cells

One of the main activities by which GC control the immune response is by regulating T lymphocyte activation, differentiation and survival. Among the genes transcriptionally regulated by GC in T lymphocytes, GILZ is rapidly induced by GC by DEX, a synthetic GC [4,43]. 

In T lymphocytes, GILZ acts mainly by inhibiting the signals under the TCR, including the MAPK-ERK, PI3K-Akt, and nuclear factor (NF)-κB pathways. In particular, GILZ inhibits NF-κB transcriptional activity by binding its p65 and p52 subunits and limiting their nuclear translocation [11,15]. GILZ also interacts with and inhibits the activity of Ras and Raf-1, components of the Ras-Raf-MAPK (MEK)-ERK pathway, one of the most important signaling cascades among all MAPK signal transduction pathways, which plays a pervasive role in controlling T cell activation and proliferation and in inflammatory cytokine production. GILZ decreases MAPK-ERK pathway activation and inhibits cell growth and proliferation [11,12,44]. This effect may be relevant in T cell growth and differentiation by regulating the downstream and/or interconnected pathways, including the NF-κB, PI3K and MAPK pathways, all involved in controlling cell growth and inflammatory processes upon TCR triggering [45]. GILZ inhibits the PI3K-Akt pathway in different cells and tissues, including lymphoid cells [46,47,48]. Inhibiting GILZ over-expression in these pathways dampens anti-CD3-induced IL-2 production, IL-2 receptor (IL-2R) expression, and Fas and FasL up-regulation, indicating that similar to GC, GILZ can inhibit T cell activation. Given the importance of NF-κB in controlling Fas and FasL and in IL-2 and IL-2R expression, this is an important indication of the molecular events responsible for the effects of GILZ on T lymphocyte activation [49,50,51]. On the other hand, antigen-induced T cell activation decreases GILZ expression levels, both the mRNA and protein level, again suggesting that GILZ hinders T cell activation and that its presence is an anergic factor for T lymphocytes. IL-2, the main survival interleukin in activated T lymphocytes, plays a role in controlling GILZ expression by repressing the transcription factor forkhead box O3 (FoxO3), which binds the GILZ promoter and up-regulates its expression [52]. GILZ expression in T lymphocytes also inhibits cell death consequent to T cell activation [12,53]. Even in this case, the inhibitory action of GILZ on NF-κB explains, at least in part, the protective effect of GILZ on T lymphocytes, as NF-κB activity promotes pro-apoptotic stimuli involved in activated induced cell death (AICD) in T lymphocytes [54,55].

**Figure 4:**
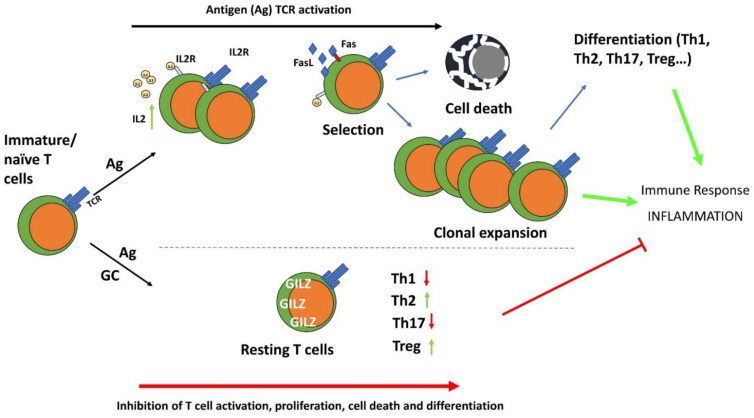
The anti-inflammatory effects of GC and GILZ in the control of T cell activation, differentiation, and survival. GC induce GILZ expression and inhibit TCR-induced T cell activation and differentiation. IL-2: Interleukin-2; IL-2R: interleukin-2 receptor; Th1: T helper type 1 cells; Th2: T helper type 2 cells; Th17: T helper type 17 cells; Treg: T regulatory cells.

After activation, T lymphocytes that survive AICD proceed to differentiate into one of the various lineages depending not only on TCR signals but also on a variety of stimuli that drive T lymphocytes into different subsets of effector helper T cell [56,57]. GC regulate T cell differentiation, suppressing Th1 cell responses and promoting Th2 and Th17 cell subpopulations, and memory T cell differentiation and maintenance [58]. Similarly to GC, GILZ promotes the switch from a Th1 to Th2 immune response; indeed, CD4+ lymphocytes of GILZ-overexpressing transgenic mice produce more Th2 (IL-4 or IL-13) and fewer Th1 (IL-1β and interferon (IFN)γ) cytokines compared to WT mice. Moreover, GILZ overexpression in T lymphocytes up-regulates GATA-binding protein 3 (GATA3) and signal transducer and activator of transcription 6 (STAT6), master regulators of Th2 switch, and down-regulates T-bet, which drives immature/naïve T cells to differentiate into Th1 lymphocytes (Figure 4) [14]. Transgenic GILZ-overexpressing mice are less susceptible to inflammatory diseases such as dinitrobenzene sulfonic acid (DNBS)-induced colitis or bleomycin-induced pulmonary fibrosis [6]. The same mice were less sensitive to an experimental model of spinal cord injury (SCI), in which GILZ inhibited the levels of pro-inflammatory cytokines (i.e., TNFα, IL-1β) and reduced the T lymphocyte activation and tissue infiltration, activated after SCI [59].

The role of GILZ as an inhibitor of immune and inflammatory responses mediated by T lymphocytes has been confirmed by studies that have detected GILZ expression in the synovium of humans with rheumatoid arthritis (RA) and of mice with collagen-induced arthritis (CIA), the severity of which is enhanced by GILZ silencing [13,60]. GILZ could be involved in regulating Th17 cell functions, inhibiting their inflammatory role and favoring their switch towards a regulatory phenotype [61], as also suggested by the transcriptomic analysis of T cell differentiation into the Th17 subset by Yosef et al., who indicated that GILZ inhibits Th17 generation and functions [62]. GILZ-dependent generation of IL-10-producing regulatory Th17 cells, and less efficient generation of Th1 and Th17 subsets implicated in autoimmune diseases have been demonstrated in a CIA model [61]. GILZ overexpression inhibits IL-17 production in salivary gland cells in a mouse model of Sjögren’s syndrome, and GILZ expression was decreased in psoriasis patients with enhanced Th17 cell activities [63,64].

GILZ is also implicated in the differentiation of Tregs, which restrain the activity of pro-inflammatory T cell subsets [43,65]. Mice with GILZ deficient T lymphocytes develop signs of loss of immune homeostasis and autoimmunity with impaired peripheral, but not thymic, Treg generation. Susceptibility to inflammation in GILZ-KO mice was exacerbated in chemically induced Th1-type colitis; GC treatment increased the Treg number and functions and alleviated the symptoms of colitis in WT mice but not in GILZ-deficient mice, suggesting that the immunosuppressive properties of GC depend, at least in part, on GILZ and involve the modulation of Treg function in vivo. Moreover, the synergistic effect of GC and TGFβ in the induction of FoxP3 and switch to a Treg phenotype is dampened in GILZ-deficient T lymphocytes [43].

The topical administration of purified GILZ recombinant protein has been used in several studies to mimic the anti-inflammatory effects of GC. GILZ protein has been delivered into the cells by different approaches; one is to fuse the cell-penetrating peptide TAT of the HIV with GILZ protein as a cell-permeable fusion protein [6,27]. GILZ recombinant protein administration mediated the suppression of experimental colitis and lethal inflammation induced by sepsis [6,25]. Furthermore, GILZ delivery to the synovial joints of mice with CIA counteracted the signs of disease to levels similar to those obtained with GC administration, confirming an immunomodulatory role of GILZ. In another mouse model of AKI, in vivo administration of GILZ protein had protective effects by inducing T cell polarization toward an anti-inflammatory phenotype [33]. The use of a GILZ-derived peptide (containing the critical amino acids 120–123, necessary for GILZ interaction with NF-κB) counteracted neuroinflammation effectively in a mouse model of multiple sclerosis [66,67]. In another study on mice with myocardial infarction, in vivo intramyocardial delivery of mesenchymal stem cells (MSCs) overexpressing GILZ, reduced inflammation during the immediate phase post-injury, associated with increased Treg number and IL-10 production, and decrease the Th17 cell subset [68]. These findings suggest that GILZ, or part of it, may have therapeutic efficacy in targeting T lymphocytes’ inflammatory effects and represents a substrate for designing of novel anti-inflammatory drugs (Figure 4).

## 7. GILZ Function in B Lymphocytes

B lymphocytes have been characterized by their ability to produce antigen-specific antibodies and to activate T lymphocytes through antigen presentation [69]. In addition, specific B lymphocyte subsets may play a critical role in regulating the immune responses through mechanisms such as cytokine production [70,71]. Impaired B lymphocyte development and function may result in harmful effects that cause inflammatory and autoimmune diseases [72], although the mechanisms controlling B lymphocyte activation and differentiation are still not completely understood.

Compared to the effects of GC on T lymphocyte functions, their role in B lymphocyte activation, differentiation and survival is less known, although many studies have demonstrated their important regulatory activity on B cells [70]. GC regulate antibody isotype switching (i.e., increased immunoglobulin (Ig)E, decreased IgG and IgA production) and synthesis, influence B cell lymphopoiesis, and suppress B cell antigenic responses [73,74,75,76]. GC have a relevant lymphotoxic effect on B lymphocytes [77,78], in particular on immature B cells, which are particularly sensitive to GC-induced apoptosis, against which mature B cells are resistant [79].

As stated earlier, GC up-regulate GILZ in a wide variety of cell types, including B lymphocytes. GILZ is highly expressed in B lymphocytes, in both the bone marrow and the spleen or other peripheral organs. In bone marrow cells, GILZ is expressed in all B cell developmental stages [80]. Similarly to that shown in T lymphocyte activation, B cell receptor (BCR) triggering inhibits GILZ expression and GILZ downregulation during activation facilitates B-cell activation [81].

A study using a GILZ-KO mouse model showed that GILZ mediates GC anti-inflammatory and immunosuppressive functions in B lymphocytes, such as growth and apoptosis, indicating that deregulated GILZ expression could be implicated in the pathogenesis of B cell disorders [80]. Moreover, GILZ-deficient B lymphocytes are partially resistant to GC-induced cell death, suggesting that GILZ could be a mediator of the pro-apoptotic effects of GC on B cell survival. Decreased B cell apoptosis in GILZ-deficient mice correlated with increased NF-κB transcriptional activity and Bcl-2 expression. 

**Figure 5:**
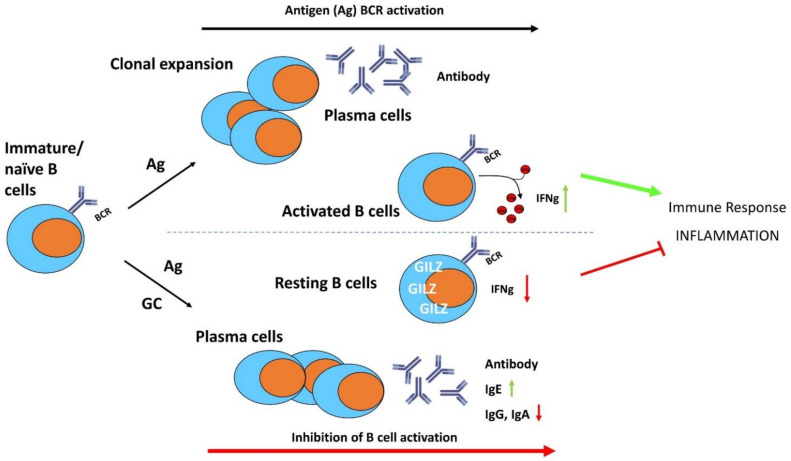
The anti-inflammatory effects of GC and GILZ in the control of B cell activation, differentiation, and survival. GC induce GILZ expression and inhibit BCR-induced B cell activation and differentiation. IFNγ: interferon gamma; Ig: immunoglobulins.

Most recently, the role of GILZ in mediating GC effects in B lymphocytes has been investigated by the generation of a conditional B cell-specific GILZ-KO mouse model (GILZ B cKO) [82]. The lack of GILZ in B lymphocytes led to increased IFNγ production in the B lymphocytes in response to inflammatory stimuli, and was associated with enhanced susceptibility to experimental colitis in mice (Figure 5). Together with the increased IFNγ production in the B cells in the GILZ B cKO mice, there was enhanced activity of the transcription factor Activator Protein 1 (AP1) on the IFNγ promoter [82]. 

Dysregulated GILZ expression could play a role in the development of certain autoimmune/inflammatory pathologies with aberrant B cell activity. Reduced GILZ expression levels were observed in the B lymphocytes of patients with Systemic lupus erythematosus (SLE), a multisystem autoimmune disease, and decreased GILZ expression in GC-treated SLE patients was associated with an increased disease activity [83].

Therefore, GILZ may regulate B cell maintenance, is expressed in resting B lymphocytes to maintain quiescence and is induced by GC to modulate the immune and inflammatory responses in activated B lymphocytes (Figure 5); GILZ deregulation could be involved in disease predisposition and could represent a diagnostic marker for predicting sensitivity and/or resistance in B cell disorders.

## 8. GILZ in Various Cell Types

Since our research group’s first attempt to produce a viable GILZ total body KO mouse, the existence of a defect in male mouse fertility was undeniable. Previous studies on cell lines and in ex vivo mouse tissues have demonstrated the pivotal role of GILZ in immune cells, but it was then quite clear that GILZ is also expressed with important functions in cells other than those of the immune system. Several studies have demonstrated that GILZ and its long isoform (L-GILZ) are critical proteins for complete spermatozoa maturation [44,84,85]. In fact, their ablation leads to infertility in male mice, but studies with the recombinant protein TAT-L-GILZ restored the normal proliferation rate in the GILZ-KO mouse airway [48]. The mechanism by which GILZ promotes germ cell survival relies on inhibiting FOXO1 nuclear translocation, with suppression of one of its target pro-apoptotic genes, i.e., *BIM* [86]. Consequently, the absence of GILZ promotes aberrant FOXO1 expression that leads to massive spermatogonia apoptosis. Elucidating the role of GILZ in spermatogenesis opens up new avenues that can lead to the therapeutic use of recombinant GILZ-based proteins for treating infertility.

GILZ is expressed in human epithelial cells and is upregulated by GC; in these cells it mediates the anti-inflammatory effects of DEX by inhibiting NF-κB [87]. Furthermore, GILZ can inhibit the MAPK-ERK signaling pathway, reducing cell proliferation and migration in airway epithelial repair [88]. Inhibiting ERK activation is also the mechanism by which GILZ stimulates transepithelial Na^+^ transport in kidney epithelial cells [89], a role controlled indirectly by aldosterone. Aldosterone regulates Na^+^ and K^+^ balance in response to environmental conditions via induction of target genes, one of which is represented by *GILZ*. GILZ controls the Na^+^-Cl^−^ cotransporter (NCC) activity in the distal nephron, which explains the observation that GILZ-KO mice have increased plasma K^+^ concentrations and lower fractional excretion of K^+^ than WT mice [90]. Aldosterone can also induce GILZ in other cell types, such as human synovial fibroblasts, in which GILZ mediates leptin production by corticoids [91]. Leptin is a pro-inflammatory adipokine involved in osteoarthritis pathogenesis. The induction of leptin by GILZ represents one of the rare conditions in which GILZ contributes to GC-dependent adverse effects. GILZ has also been detected in RA synovial fibroblasts and is highly expressed in the synovium of patients with active RA. Interestingly, GILZ overexpression in synovial fibroblasts inhibited IL-6 and IL-8 release, exerting beneficial anti-inflammatory effects. GILZ was detected in the synovial sublining and endothelium of RA patients at higher levels than in healthy tissues [60]. In these cells, GILZ reduces the expression of adhesion molecules, cytokines and chemokines, to prevent leukocyte transmigration, by inhibiting NF-κB and MAPK activation, further dampening inflammation [92]. In other endothelial cell types, specifically rat primary retinal microvascular endothelial cells (RMECs), GILZ overexpression reduced the expression of adhesion molecules such as intercellular adhesion molecule 1 (ICAM-1), and attenuated NF-κB p65 nuclear translocation, exerting an anti-inflammatory effect [93]. Similarly, the implication of GILZ in inflammatory processes has been shown in other endothelial cells, specifically in degenerated aortocoronary saphenous vein bypass grafts, in which GILZ expression levels were decreased compared to those in healthy veins, suggesting its involvement in cardiovascular disease [94]. The importance of GILZ in the cardiovascular system is proven by its expression in cardiomyocytes, in which it mediates the cytoprotective effects of GC during the treatment with the chemotherapeutic agent doxorubicin [95]. Moreover, GILZ involvement in cardiovascular diseases was very recently observed in an angiotensin-induced model of hypertrophy and diastolic disfunction in mice. In that experimental model, GILZ-KO mice showed enhanced cardiomyocyte hypertrophy with normal myocardial inflammatory and fibrotic reaction, suggesting that GILZ is involved in hypertrophic growth but not in local inflammation [96]. Furthermore, in a mouse model of heart infarction, reduced cardiac GILZ correlated with strong immune and inflammatory responses, whereas intramyocardial GILZ delivery increased Tregs and IL-10+ cells, while reducing Th-17 lymphocytes with subsequent cardioprotection [68].

Therefore, in some cell types and under specific conditions, GILZ mediates the anti-inflammatory effects of GC, but in others it can have either different functions from those of GC or even mediate some adverse effects of GC. Another example is the anti-myogenic role of GILZ in myoblasts. GILZ/L-GILZ over-expression resulted in reduced myotube formation and the inhibition of myoblast differentiation, accounting for the anti-myogenic effect of GC. Importantly, both isoforms inhibited myogenic differentiation 1 (MyoD) transcription, one of the most critical muscle-related factors [97]. Another study correlated this anti-myogenic function of GILZ to statin-induced myopathy, representing a toxic effect of statins. Statins induce FOXO3, a transcription factor that promotes GILZ transcription, situating GILZ as a critical mediator of statin-induced muscle damage [98].

As an index of inflammation, the evaluation of GILZ expression was revealed to be of great importance due to the correlation of poor GILZ expression with the inflammatory status. As an example, the aqueous humor of the eyes of patients with bacterial endophthalmitis had lower GILZ expression levels than that of people with age-related cataracts. Accordingly, in the experimental model of LPS-induced retinal inflammation in rats, GILZ overexpression suppressed the production of cytokines such as IL-1β and IL-17, partially overlapping with the anti-inflammatory effects of GC [99]. Interestingly, in the same tissue, i.e., the retina, GILZ not only exerted an anti-inflammatory effect, but was also neuroprotective by attenuating light damage-induced photoreceptor apoptosis. GILZ overexpression causes the upregulation of the anti-apoptotic protein Bcl-xL and the downregulation of caspase-9 and caspase-3, which are activated by light injury [100]. A similar mechanism has been observed in cochlear cells, in which GILZ was protective against endoplasmic reticulum stress-induced apoptosis. GILZ Overexpression decreased the cleaved form of caspase-3 and Bax, whereas it increased Bcl-xL expression, contributing to apoptosis prevention in cochlear cells [101].

An organ that is particularly interesting in terms of GILZ expression is the brain. Yachi and coworkers assessed the presence of GILZ and reported abundant GILZ expression in brain and spinal cord neurons, with localization to the piriform cortex, globus pallidus, anterior amygdaloid area, thalamus and motor neurons, whereas low GILZ expression levels were localized to neurons of the caudate putamen, cerebral cortex and hypothalamus [102]. The study of GILZ function in the brain is still ongoing, but it seems to be related to the neuroprotective and neuroinflammatory responses to GC [103]. Interestingly, one study demonstrated GILZ as a possible candidate for predicting changes in hippocampal volume, such as those observed in major depressive disorder [104]. One important function has been studied in the chicken embryonic pituitary gland, in which GC upregulates GILZ to control the expression of hormones in the developing neuroendocrine system [105].

Adipocytes are another cell type in which GILZ plays a specific role by antagonizing the pro-differentiation role of GC. Adipocyte accumulation in the bone marrow is a detrimental effect of GC treatment, resulting in high blood pressure, and collapse of vessels with consequent osteonecrosis in the femoral head. On the other hand, GC are required for osteogenic differentiation. This dual role of GC can be explained by the opposite effects of their target genes. As an example, GILZ inhibits the transcription of peroxisome-proliferator-activated receptor-γ2 (PPARγ2), a protein that promotes adipogenesis, thereby antagonizing GC effects in MSC [106]. This inhibition favors MSC commitment towards the osteogenic lineage; therefore, GILZ mediates the endogenous GC-induced anabolic effect on osteogenesis [107,108,109].

A function of GILZ has also been discovered in the liver. Patients with alcoholic hepatitis (AH) have lower GILZ levels than non-AH alcoholic patients. In particular, GILZ was expressed in the Kupffer cells of all AH patients, more predominantly than in healthy people [110]. Furthermore, GILZ deficiency favors liver fibrosis development, a feature shared by several liver pathologies, including alcoholic liver cirrhosis and autoimmune hepatitis. Accordingly, patients with liver fibrosis have low liver levels of GILZ. As GILZ downregulates C-C motif chemokine ligand 2 (CCL2) expression, macrophages and CD4+ T cell recruitment in the liver is restrained. Therefore, the GILZ-CCL2 axis may represent a potential target for discovering new therapeutic agents for treating liver fibrosis [111].

The direct involvement of GR and GILZ has been found in the increased release of pro-inflammatory cytokines in the liver of obese mice. In their Kupffer cells, GR and GILZ expression levels were reduced, contributing to the inflammatory state in obese mouse liver [112].

Cancer development encompasses complex molecular events that are still far from being fully explained. Given that inflammation and inflammatory players, such as cytokines, are among the multiple contributors to the tumor microenvironment, GC are widely used for treating some cancer types by virtue of their anti-inflammatory and immunosuppressive effects. As one of the most important GC-induced genes, *GILZ*, has been studied in tumoral experimental conditions to unravel its possible involvement in tumor physiology and pharmacology. In particular, L-GILZ has been implicated in the control of tumor growth, due to its ability to bind MDM2 and p53, rendering p53 available as a tumor suppressor protein [113]. In addition, L-GILZ overexpression in a human thyroid cancer cell line inhibited cell growth both in vitro and in xenograft transplantation in mice [114,115,116]. Conversely, GILZ also promoted tumor growth, as it is highly upregulated in tumor microenvironment DC, suppressing the immune T cell response against cancer [117]. Therefore, based on the cells in which they are primarily expressed, GILZ and L-GILZ can control tumor growth and development via either suppression or promotion, representing a potential target or tool for cancer treatment.

From the numerous studies on diverse cell types, GILZ has emerged as a hallmark of either GC treatment success or a mediator of GC-derived physiological effects, in addition to the unique properties and functions of GILZ protein. At the same time, GILZ can be increased or decreased in several pathological conditions, becoming a possible marker of disease. In rare conditions, GILZ can also mediate GC adverse effects, as described earlier.

## 9. Conclusions

Components of the endocrine system regulate many stages of inflammatory and immune response. Stress increases neuroendocrine hormones, particularly GC and catecholamines but to some extent also prolactin, growth hormone and nerve growth factor. Stress, via the action of hormones, can induce detrimental effects on the immune and inflammatory responses, including reduced natural killer cell activity, lymphocyte populations, lymphocyte proliferation and survival, and antibody production, influencing infections, and tumor growth, but also the pattern of inflammatory and autoimmune diseases. Notably, hormones can act as modulators acting directly and/or indirectly on the inflammatory/immune response and some exert a direct anti-inflammatory effect, such as with GC.

Endogenous GC, released by the adrenal gland and other concentrations by different organs, regulate many functions of virtually all body organs including the inflammatory/immune processes. Most of their effects are due to transcription regulation and some endogenous proteins have been identified as mediators generated consequent to the GC-GR interaction, such as Anxa-1 and GILZ. GILZ was firstly discovered as an endogenous protein that could interfere with T cell immunity and consequently modulate the immune and inflammatory processes through interference with intracellular mediators, including NF-κB, AP-1, and the MAPK complex. Further studies have proven the ability of GC-induced GILZ to regulate the activity of other components of the inflammatory burden including macrophages, DC, neutrophils and B lymphocytes.

Of particular interest is the possible relationship between the two major effectors of GC, ANXA-1 and GILZ. In fact, how GILZ is important for the GC-induced production of ANXA-1 has been demonstrated, in that it regulates ANXA-1 expression at a transcriptional level, but ANXA-1 also regulates GILZ expression.

**Figure 6:**
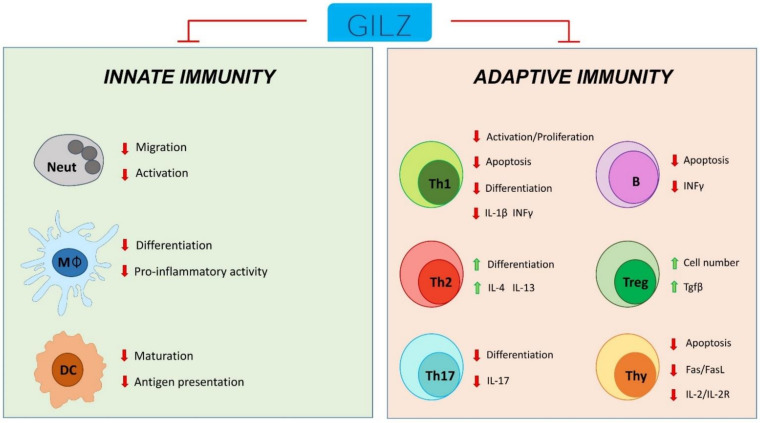
Schematic representation of GILZ participation in the control of innate and adaptive immune cell responses. Neut: neutrophils; Mφ: monocytes/macrophages; DC: dendritic cells; Th1: T helper Type-1 cells; Th2: T helper Type-2 cells; Th17: T helper Type-17 cells; Treg: T regulatory cells; Thy: thymocytes; B: B cells.

Therefore, GILZ can be considered both a player acting on behalf of GC but avoiding their unwanted adverse effects and a marker of a resolved inflammatory state (Figure 6).

In the future, tools comprising GILZ-based peptides could be useful for treating chronic inflammatory diseases, and GILZ detection could become a diagnostic strategy for evaluating the efficacy of a specific pharmacological treatment.

## Data Availability

Not applicable.
